# Analysis of risk factors for maxillary denture-related oral mucosal lesions: A cross-sectional study

**DOI:** 10.4317/medoral.22826

**Published:** 2019-04-24

**Authors:** Michele F. Brantes, Rebeca S. Azevedo, Rafaela E. Rozza-de-Menezes, Helvécio C. Póvoa, Renata Tucci, Adriele F. Gouvêa, Ademar Takahama-Jr

**Affiliations:** 1 DDS, MSc; Departamento de Formação Específica, Universidade Federal Fluminense (UFF), Nova Friburgo, Rio de Janeiro, Brazil; 2 DDS, PhD; Departamento de Formação Específica, Universidade Federal Fluminense (UFF), Nova Friburgo, Rio de Janeiro, Brazil; 3 PhD; Departamento de Formação Básica, Universidade Federal Fluminense (UFF), Nova Friburgo, Rio de Janeiro, Brazil; 4 DDS, PhD; Departmento de Medicina Oral e Odontologia Infantil, Universidade Estadual de Londrina (UEL), Rua Pernambuco 540, Londrina, Paraná, Brazil

## Abstract

**Background:**

To evaluate the frequency of maxillary dentures-related lesions and the possible associated risk factors.

**Material and Methods:**

Ninety-seven participants were selected, and a complete anamnesis, physical examination and tests of occlusion vertical dimension (OVD), retention and stability of the denture, biofilm quantification, cytopathology, sialometry, pH analysis and buffer capacity of the saliva were performed. Statistical analyses were performed with the Pearson’s chi-square, Mann-Whitney tests, and Pearson’s coefficient (*p*<0.05).

**Results:**

In 78% of the participants at least one denture-related lesion was found. Denture-associated stomatitis (63%), inflammatory fibrous hyperplasia (19%) and traumatic ulceration (11%) were the 3 most frequent lesions. The habit of night use of the denture was considered an independent risk factor for the development of oral lesions [OR=3.0 (95% CI 1.09-8.56); *p*<0.05]. Furthermore, the longest period of use of the same denture and biofilm also had statistically significant relation to oral lesions. The biofilm seems to be more related to the prevalence of oral lesions according to the multiple logistic regression [OR=1.3 (95% CI: 1.01-1.83) *p*<0.05]. The lack of a dentures’ cleaning solution and detrition of the prothesis were independent risk factors for denture-associated stomatitis. Male gender, loss of OVD and bad buffer capacity were risk factors for angular cheilitis. Fractures of the base and repair of broken dentures were risk factors for traumatic ulcers.

**Conclusions:**

These results show a high frequency of denture-related lesions. Besides, participants hygiene habits and poor quality of the dentures were the main factors for the development of these lesions.

** Key words:**Complete denture, oral health, oral hygiene, oral lesion, saliva.

## Introduction

The proportion of elderly is increasing worldwide, as well as the concern about the oral health status of this population. As the life expectancy of the population increases, it also increases the number of individuals requiring dentures (1). The prevalence of removable prosthesis, especially dentures, is high among the elders, especially among people with low socioeconomic status (2). Even nowadays, most of the people with total edentulism continues to receive conventional prosthetic treatment, instead of oral implants (3).

Although the removable prosthesis represents a good rehabilitation option in cases of edentulism, maintenance and monitoring are mandatory. Complete dentures require special hygiene and maintenance care, since poor denture hygiene is associated to oral and systemic diseases (4). Therefore, recall visits and shortened denture usage would facilitate the prevention of the oral mucosal lesions and the maintenance of oral hygiene, thus improving the quality of life for the denture wearers (5).

The main mucosal lesions associated to removable prosthesis are denture-associated stomatitis, angular cheilitis, inflammatory fibrous hyperplasia, and traumatic ulcers. The presence of candida infection, poor retention and mechanical trauma have been associated with the development of these lesions (6). In addition, low salivary pH, reduced OVD and residual ridge resorption may also be associated with denture-associated stomatitis, angular cheilitis and traumatic ulcer, respectively (7).

Saliva is known to play an important role in the retention of dentures, acting as a molecular link between the mucosa and the denture base (8). Older people frequently present a decrease in the salivary flow rate (hyposalivation) as a cause of dehydration, drugs or diseases (9). The hyposalivation may lead to sensitive points in the mucosa, lack of retention and contributes to the development of mucosal lesions (10). Some salivary parameters such as salivary flow and pH are related to one another, and a reduction in salivary flow generates a significant decline in oral defense systems, which can cause caries and oral mucosa inflammation (11).

Therefore, the present study was carried out to evaluate the relative frequency of maxillary denture-related lesions and to determinate the main risk factors associated to this process.

## Material and Methods

This study used a cross-sectional design and was conducted in a period of two years. The study population consisted of 97 maxillary denture wearers participants attended at Instituto de Saúde de Nova Friburgo of Universidade Federal Fluminense, Brazil. Participants who were underage or legally unable to understand and sign the informed consent form were excluded from this study. The informed consent was obtained from all voluntary participants and the study protocol was approved by the local ethics committee (number 1.145.135). Standardized questionnaires were applied for demographic data (age, sex, education level), medical history (pre-existing diseases and drugs in use) and information about the prosthesis: denture hygiene (immersion on cleaner solution), period of use of the same denture and the habit of night use. After the questionnaire, clinical tests were carried out and the physical examination of the mucosal and the prosthesis was performed by a calibrated examiner. A previous training of the examiner, including review of criteria and protocol, demonstrations examinations and practice examination together with the responsible researcher was carried out for calibration. Biopsies were performed in cases that needed histopathological analysis to confirm the diagnosis. Participants with denture-associated stomatitis were ranked according to the degree of stomatitis recommended by Newton (12) in type 1, 2 and 3: Type 1 - scattered spot areas of the palatine mucosa inflammation dispersed throughout the normal mucosa; Type 2 - palatal mucosa presenting as generalized inflammation in the area covered by the prosthesis; Type 3 - hyperemic palatal mucosa presenting nodular appearance.

-Clinical tests:

The evaluation of the OVD was performed using the combination of aesthetic, metric and phonetic methods. In the aesthetic method, references of satisfactory OVD were: the conformation of nasolabial folds, the harmony of the lower face with the other parts of the face and facial fullness consistent with the patient’s age. In the metric method, a Willis gauge was used to obtain the difference between the OVD and resting vertical dimension (RVD) corresponding to the freeway space, considered normal minimum of 3.0 mm. In the phonetic method, patients were asked to pronounce words with sibilant sounds such as “Mississippi” and “sessenta e seis” (sixty-six) while the movement of the jaw was watched, forming an interocclusal space called free functional space, being considered normal OVD when a minimum space of 0.5 to 1.0 mm was obtained (13). The retention of the maxillary denture was evaluated by the examiner placing the index finger and thumb in the premolar areas and exerting a gentle downward vertical pressure. The stability was evaluated by the examiner placing his index fingers and thumbs on both sides of the premolars and applying lateral and rotational forces. The retention and stability were satisfactory in the absence of displacement or scale movement of the denture (14).

For the biofilm quantification, the prostheses were photographed with a Canon T3i with macro lens Canon 100 mm 2.8 USM AF and circular flash Canon MR-14 EX coupled to the wide-field fluorescence equipment that emits blue / violet light at 400-460 nm wavelengths. With this dispositive, the biofilm fluoresces in a bright red color due to the presence of endogenous porphyrins, a phenomenon that can be used to quantify plaque *in vivo* or *in vitro* (15). The prosthesis of each palate surface was divided into four sections of approximately equal areas by mentally drawing a line anteroposteriorly at the midline and another line perpendicular to the midline at about the premolar region. Each quadrant was rated as 0 = no plaque; 1 = light plaque (25% or less covered quadrant); 2 = moderate plaque (26% to 50% covered quadrant); 3 = large quantity of plaque (51% to 75% of covered quadrant); and 4 = very large quantity of heavy plaque (76% to 100% of covered quadrant). The total score was obtained by adding the scores of the quadrants, the maximum score was 16 (16). The prosthesis also was evaluated for the absence or presence of: loss and/or fracture of teeth, detrition, cracks, resin porosities, fractures of the base, irregular borders, reassembly, repairs and suction chamber.

The cytopathology examination was performed on the palate and in the prosthesis to evaluate the presence of Candida sp. The hard palate mucosa and the internal surface of the prosthesis were scraped with a cytobrush device applying pressure and rotation. The material was immediately smeared on a clean glass slide and fixed in 99% ethanol. Slides were stained with periodic acid of Schiff to highlight the presence of the fungal hyphae and microscopically evaluated.

The salivary flow rate at rest and the stimulated salivary flow rate were measured by sialometry. The participant was instructed to keep the mouth half-opened and the saliva passively flowed to a sterile tube positioned near the mouth. For the measurement of salivary flow rate at rest, the patient was instructed to swallow all the saliva present in the mouth and saliva was collected for 5 minutes without stimulus. For the stimulated salivary flow rate, the patient was instructed to chew a mechanical sialagogue for 1 minute and after that time swallowing the produced saliva. Subsequently, the participant was asked to chew the device for 5 minutes, depositing the saliva produced in the collector tube.

The amount of saliva collected in both situations was measured separately using a 10mL disposable syringe determining the salivary flow measure (mL/minute). The values were classified according to Nederfors (17) in rest: normal ≥ 0.3; low ≥ 0.1 and < 0.3; very low < 0.1; Stimulated: normal ≥ 0.7; low < 0.7 - 0.5; very low < 0.5. The measurements for pH and salivary buffering capacity were performed after the clinical care of participants.

The pH was measured with the aid of a pH indicator strip - 0 to 14 (Machar Nagel ®), by the colorimetric method and was considered normal between the values of 6.5 - 7.5. The buffer capacity analysis was performed by mixing 500 µL of saliva with 1.5 mL of HCl (hydrochloric acid) to 5 mM in a sterile microtube. The wells were, then, shaken for 1 minute and opened for 5 minutes to output the CO2, after this, 10 µL was pipetted onto the saliva pH indicator strip - 0 to 14 (Machery Nagel®) and the result was read immediately (18). The classification of the buffer capacity was performed based on the final pH according to Cavasin and Giovani 18 in: great > 5.6; regular: between 4.5 and 5.5; bad <4.5.

-Statistical analysis:

The relationship between the variables according to the presence of denture-related lesions were examined with Chi-square or Mann-Whitney test. Pearson’s correlation coefficient tests were applied to determine the correlation between the quantitative variables. The presence or absence of oral lesions (binary variables) was firstly analyzed using univariate test (Chi-square) to determine the risk factor. Then, we included only data with statistically significance in the multiple logistic regression to achieve simultaneous observation about risk factors for more than one outcome variable. *P*-values <0.05 were considered statistically significant. A risk factor is defined as any characteristic that increases the likelihood of developing the lesion.

## Results

-Participants and prothesis data

From the 97 participants, 63 (65%) were women and 34 (35%) were men. The mean age was of 63 years, varying from 44 to 86 years (±8,6), and the schooling average was 6 years. Ninety-two participants (95%) reported the continuous use of some medication, mainly antihypertensives, antidiabetics, statins and antisecretors.

The period of use of the same denture ranged from 2 months to 60 years, with a mean of 14 years. The participants reported to clean the dentures 3 times a day, in average. 63% of the participants reported to use the dentures during the night, and 31 (32%) reported leaving the dentures immersed in some solution overnight, most commonly water or a mixture of water and sodium hypochlorite.

**Figure 1:**
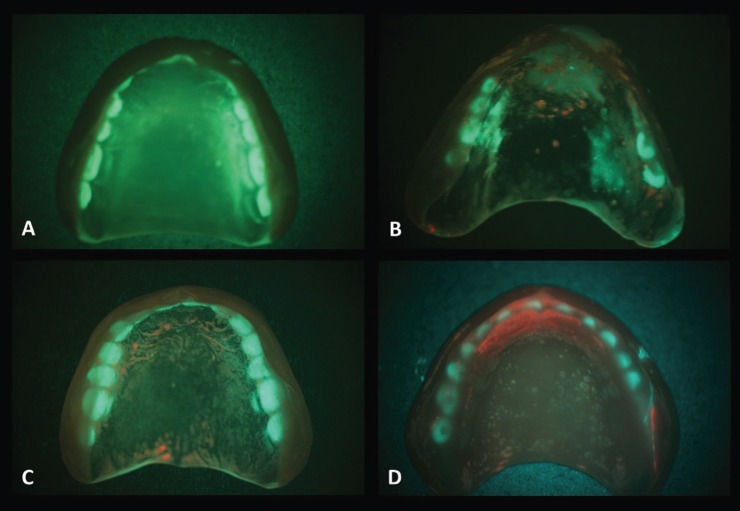
Biofilm quantification in the dentures. Examples of scores: A – 0, B – 4, C – 6 and D - 10.

According to the factors related to the prosthesis status, most of participants had unsatisfactory OVD (49%), unsatisfactory stability (69%) and satisfactory retention (60%). All evaluated dentures presented some irregularity, in descending order: resin porosities (97%), detrition of resin and/or tooth (77%), irregular borders (76%), cracks (44%), artificial teeth loss or fracture (29%), repairs (29%), fractures of the base (18%), reassembly (8%) and suction chamber (3%). Regarding the biofilm classification by wide-field fluorescence, the average score was 3 points, varying from 0 to 14 points, and only 11 cases (15.9%) were classified with score 0 (Fig. 1).

-Oral lesions evaluation

Most participants (78%) presented some denture-related lesion. Among them, 53 (70%) were women and 23 (30%) were men. Lesions found in order of frequency were: denture-associated stomatitis (63%) affecting the palate and residual alveolar ridge; inflammatory fibrous hyperplasia (19%), affecting mainly the anterior alveolar ridges and including 3 cases of suction chamber hyperplasia. Traumatic ulceration was found in 11 cases (11%), mainly in the alveolar ridge region; 8 cases (8%) presented angular cheilitis; and two cases frictional keratosis in the anterior alveolar ridge and palate. Of the 61 participants with denture-associated stomatitis, 25 (41%) presented lesions of type 1, 25 (41%) of type 2 and 11 (18%) of type 3.

-Analysis of the saliva

Most participants presented hyposalivation considering the salivary flow rate at rest (54%). Considering the stimulated salivary flow rate, 55 (56%) of the participants had normal salivary flow, 17 (18%) had low salivary flow and 25 (26%) had very low salivary flow. The mean pH was 7.3 (± 0.6), and only 4 (4%) participants had pH values below 6.5. The mean buffer capacity was 5.5 (± 1.0), considered regular. Of all participants, 45 (46%) had optimal buffer capacity, 37 (38%) regular and 15 (16%) poor. Patients who had a normal resting salivary flow had also under stimulation (*p* <0.0001) and those who had higher salivary pH values had also better salivary buffering capacity (Pearson correlation test, *p*<0.0001 for both cases).

-Cytopathology analysis

In 69 cases, it was possible to perform cytopathology examination to evaluate the presence of candida hyphae. From this sample, in 14 cases (20.3%) it was possible to identify the fungus in the palate and in 32 cases (46.4%) in the inner surface of the prosthesis. In only 11 cases (15.9%), the fungus could be identified in both the prosthesis and the palate.

-Results of Statistical Analysis

In the univariate analysis, the presence of any denture-related lesion was associated with the habit of night use [OR: 3.0 (95% CI: 1.09- 8.56); *p*<0.05], the longer the period of use of the same denture (Mann-Whitney test; *p*<0.05) and biofilm accumulation (Mann-Whitney test; *p*<0.05). The analysis of the adjusted residual showed that the prevalence of oral lesions in participants wearing their dentures during the night (83.6%) was 2.2-fold higher than in individuals without this habit and without oral lesion (37.5%) and in individuals not immersing the dentures (84.8%) was 2.3-fold higher than in those with this habit and without oral lesion (35.5%) ( Table 1).

**Table 1:**
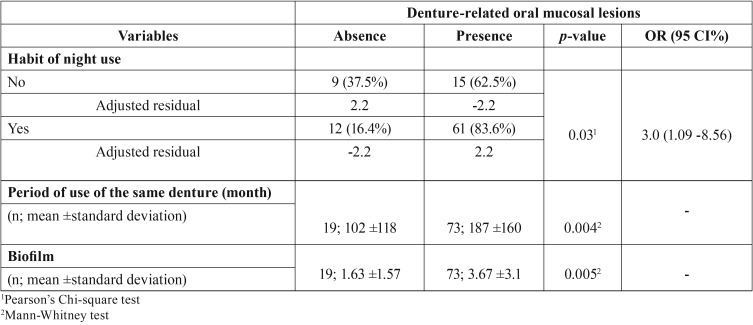
Data association with presence of the denture-related oral mucosal lesions.

Regarding the presence of any denture-related oral mucosal lesions and the variables with statistical significance, a multivariate logistic regression (binary) was performed showing that only the presence of biofilm in the denture surface seems to influence their development [OR=1.3 (95% CI: 1.01-1.83); *p*<0.05].

The relative frequency of denture-associated stomatitis was associated with detrition and lack of immersion ( Table 2). The presence of angular cheilitis was associated with male gender, loss of OVD and bad buffer capacity ( Table 3). There was a reduction of approximately 86% of the angular cheilitis in individuals with good buffer capacity.

**Table 2:**
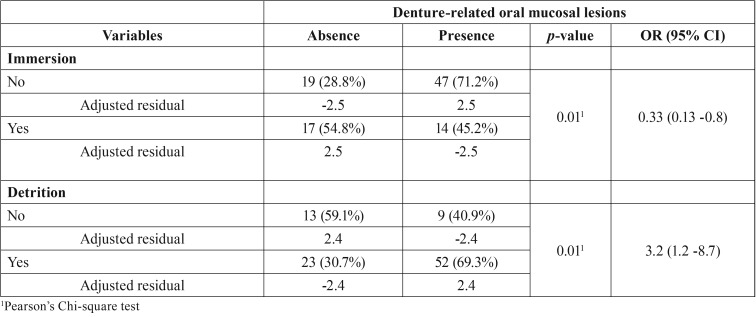
Variables significantly associated with denture-associated stomatitis.

**Table 3:**
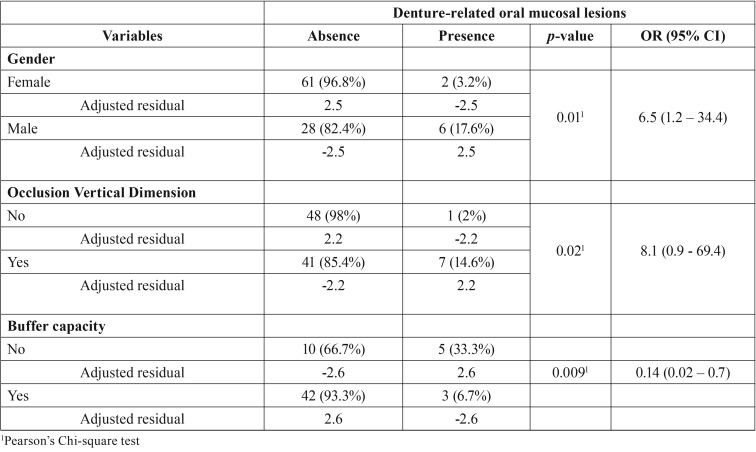
Variables significantly associated with angular cheilitis.

Comparing the results of the cytopathology analysis with the presence of denture-associated stomatitis, from the 49 cases with stomatitis only 8 had the hyphae detected in the palate and 24 in the prosthesis. In the 20 cases without denture-associated stomatitis, 6 had the hyphae detected in the palate and 8 in the prosthesis. However, this proportion was not statistically significant. In all 4 participants with angular cheilitis, it was possible to detect the hyphae in the mucosa or in the denture.

The presence of traumatic ulceration was associated to the presence of fractures in the base of the dentures, previous repair of broken denture and resting sialometry values ( Table 4). No variable was statistically associated to the presence of fibrous hyperplasia.

**Table 4:**
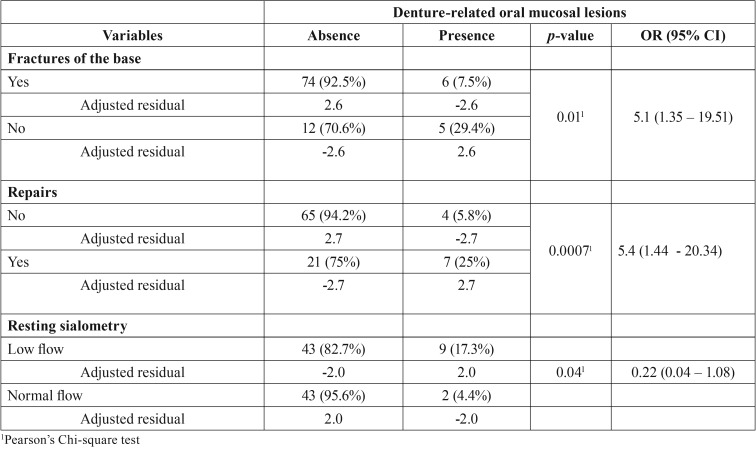
Variables significantly associated with traumatic ulcer.

The Pearson correlation test showed that the longer period of use of the same denture, the higher the biofilm accumulation (*p*<0.05) and also the higher the denture-associated stomatitis degree (*p*<0.05). The values of the salivary flow rate at rest were directly proportional to the values of the stimulated salivary flow rate (*p* <0.0001) and the higher the pH the higher the buffer capacity (*p* <0.0001).

There was no association between the values the salivary flow rate at rest and the stimulated salivary flow rate with retention of the prosthesis. However, patients with satisfactory stability had higher values of the stimulated salivary flow rate than those with unsatisfactory stability (*p*<0.05).

## Discussion

The use of complete maxillary dentures is still very common in our population, especially among elderly. It is a very good kind of oral rehabilitation but can induce several mucosal damages when not used properly. The use of dentures is associated with a high frequency of oral lesions, varying from 50 to 75% (5,6,19-20). In the present study, 78% of the participants presented some lesion associated with maxillary dentures. Also in according to most of the studies, the frequency of these lesions was higher in female participants (70%) (19,20). The reason for the high frequency of these lesions in women is still unclear, it is suggested that it is because women seek more prosthetic treatment and use their prosthesis for prolonged periods without removal for aesthetic purposes (21); in this study, from the 63 women, 39 (62%) used their prosthesis without removing during the night. Other authors suggest that a mucosal atrophy of the oral cavity may occur during or after menopause, in this way, the atrophic oral epithelium offers little protection against various irritants and is therefore more susceptible to the development of hyperplasia as a response to chronic irritation from poorly fitting prostheses (22).

The poor oral conditions of people using total dentures generate negative impact on their life quality. The lack of education and poor hygiene of the dentures in the elders interfere with general health and are an important public health problem (1). Poor oral health is directly associated to systemic health, the use of the prosthesis during the night has been reported to confer a two-fold increased risk of severe pneumonia by aspiration in the elders (4). In our study, the biofilm was an important risk factor to the development of a denture-related lesion according to the multiple analysis. Considering this fact, social centers for the elderly, community dentists and other health professionals are required to provide education on oral health, hygiene of oral prostheses, care habits, the importance of regular recalls and promote preventive dental programs for this specific population (23).

A combination of mechanical hygiene through brushing with chemical agents is a good choice for patients with dentures (24). Chemical agents with potential for bacterial growth inhibition are 1% sodium hypochlorite, vinegar and chlorhexidine digluconate and may be considered suitable products for cleaning dental prostheses (25). Immersion in sodium hypochlorite for 10 minutes once a week does not appear to impair the acrylic alloys of a denture (24). The use of chemical agents might be a good strategy for denture cleansing to be performed by patient or a caregiver, as a part of an oral health program.

Moskona and Kaplan reported that all denture-related lesion frequencies increased with patient age and the longer period of use of the same denture. In this study, the presence of lesions was also associated with the longer period of use of the same denture. The deterioration of the denture by the time of use causes wear of the acrylic, irregular edges besides surface rough and with cracks. This increases the chances of the patient developing lesions such as fibrous hyperplasia, denture-associated stomatitis, angular cheilitis and traumatic ulcers (26). Older patients are often reluctant to restore or replace their dentures. After 2 years of use of the denture, the acrylic teeth suffer wear and vertical height loss is clinically relevant, therefore an evaluation must be made for possible replacement (27).

Denture-associated stomatitis was the most frequent lesion (63%) and was related to the denture detrition, other authors also associate the use of worn and inadequate prostheses as a risk factor for this lesion (28). In addition, other factors may be related, such as smoking, longer period of use of the same denture and continuous day and night use (29).

Candida is able to adhere to the acrylic of the prosthesis, this represents the first step in the infectious process, and then this biofilm formation can lead to lesion of the adjacent mucosa (30). Tigmotropism is a pathogenic mechanism of candida. Studies show that the hypha form can invade the cracks and protuberances on the resin, the adherence of hyphae is greater on rough surfaces when compared to smooth surfaces (31). However, it was not found in this study association of denture-associated stomatitis with the presence of fungus in the dentures or palate, that sustaining the hypothesis that this lesion may be more related more to bacteria present in the biofilm of the dentures than with the fungal presence (32).

Among the denture-related lesions, the traumatic ulcers have a frequency rate ranging from 4 to 26% (32), like the frequency found in this study (11%). Factors associated with the presence of traumatic ulcerations were the fractures of the denture base, the lack of stability and the presence of previous repairs. This type of lesion can occur more often in the first days after the insertion of new prostheses and may also be associated with the use of dentures with overextension or in patients with unbalanced occlusion (33).

The frequency of angular cheilitis (8%) was similar to other studies (18). This lesion may have several predisposing factors, such as reduction of OVD, presence of chronic oral candidiasis, deficiencies of B vitamins and anemia (33). Martori *et al.* found association of angular cheilitis with the presence of Candida, lack of stability of the prosthesis and complete edentulism. The edentulous patient’s lower third of the face loses its height, and if this height is not recovered by a good denture, it tends to form folds at the angles of the mouth where there will be accumulation of saliva and consequently the skin will become fissured and secondarily infected (33). In our study, the presence of angular cheilitis was also related to loss of OVD. On the other hand, we should disregard the risk seen in the loss of OVD because the value of 95% CI included the number 1. Further studies should be performed with a larger sample of individuals with dentures and angular cheilitis. In addition, all patients evaluated by cytopathological scraping with angular cheilitis showed the presence of candida in the palate or in the denture surface. Coelho *et al.* reported a higher frequency of angular cheilitis in male patients. In this study, it was also found association between male gender and angular cheilitis (19).

Most of the participants of this study presented hyposalivation. Salivary flow is usually reduced in elders compared to young patients and is significantly more prevalent in smokers and in patients using medications, such as antihypertensives (34). Different studies have shown that hyposalivation may cause increased sensitivity of the oral mucosa, resulting in a greater risk of changes and lesions (9). Besides, adversely affects oral functions and the patient’s overall satisfaction with his or her dentures (34). Other studies show that saliva is one of those responsible for adherence, cohesion and surface tension which provide retention of a prosthesis (6). Arslan *et al.* reported that edentulous patients, who use their dentures for many years and with disturbance in salivary flow, will have less stability of the denture and consequently will present difficulties in speech and reduction in the quality of life (35).

Saliva has also important biological properties, being responsible for maintaining a relatively stable pH through its ability to act as a buffer against acids produced by microorganisms or ingested through diet (36). In this study, the mean pH was similar to a study carried out in Mexico, where the elderly, fully edentulous and with dentures had an average pH of 7.7 (37). Similar values were also reported in China, where the mean pH of patients with prosthesis was 7.2 (38). The mean buffer capacity of this study was considered regular, but higher than the average buffer capacity reported by Islas-Granillo *et al.* of 4.2. And lower than reported by Chang *et al.* of 6.9. In our study, participants with bad buffer capacity developed more angular cheilitis compared to participants with good buffer capacity, and there was no significant association between pH and the presence of denture-related lesions. Authors suggest that these salivary parameters may be influenced by the edentulous oral environment and that tooth loss can lead to functional deficiencies in the oral mucosa, oral musculature and salivary glands (39). As also described by Chang *et al.* a positive correlation was found between pH and buffer capacity in our sample.

Considering all these dentures associated problems in the elderly population, dentists are responsible to give more detailed information to the patients or caregivers about the proper care of dentures. Among this information we highlighted: 1 - Even with proper maintenance, a professional evaluation of the dentures for adjustments or possible replacement should be performed every two years; 2 - Dentures should be brushed daily, removed during the night and immersed preferably in a denture cleaning solution; and 3 - Consult a dental professional if dentures become cracked, broken or cause mucosal irritation. These guidelines should also be the focus of health promotion and disease prevention programs, once the correct maintenance of the dentures can extend their usable life and contribute to the oral and general health.

A cross-sectional study has some limitation, once the investigator measures the outcome and the exposures in the study participants at the same time. However, it can be used to measure the prevalence of disease or calculate the OR as a measure of association (40). This methodology has been the main type used to evaluate the risk-factors for denture-related oral lesions (5,6,7,20).

Within the limits of the study, it can be presumed that biofilm is considered an important factor in the development of denture-related oral mucosal lesions and the use of dentures during sleep and the lack of daily immersing the denture in cleaning solution are associated with oral lesions, mainly with denture-associated stomatitis. Further, the male gender, loss of OVD and bad buffer capacity are associated with angular cheilitis. Moreover, fractures of the base and stability of the dentures and repair of broken prothesis are associated with traumatic ulcers. Considering the systemic consequences of poor oral health and the great number of patients wearing dentures in our country, a special attention should be given by clinicians and public health officials to these denture related problems.
